# Traumatic symptoms and institutional support expectations among psychiatry residents dealing with patient suicide

**DOI:** 10.1192/j.eurpsy.2024.1664

**Published:** 2024-08-27

**Authors:** I. Yaich, C. Ben Said, T. Ben Hamida, N. Bram

**Affiliations:** ^1^Forensic Psychiatry Departement, Razi Hospital, La Manouba; ^2^Faculty of Medecine of Tunis, Tunis El Manar University, Tunis, Tunisia

## Abstract

**Introduction:**

Adult and child psychiatry residents encounter unique stressors in their training distinct from those in other medical specialties. Patient suicide has been identified as one of the most distressing experiences during psychiatric training.

**Objectives:**

This study represents the first Tunisian investigation aiming to assess (1) the impact of patient suicide on psychiatry residents and (2) the limitations of the institutional support system in dealing with such cases.

**Methods:**

A Google Forms questionnaire was distributed via email to all residents, gathering socio-demographic data, assessing traumatic impact using the PTSD Checklist for DSM-5 (PCL-5), and soliciting open-ended responses regarding personal experiences and expectations of the institutional support system.

**Results:**

Fifty-three residents participated in the study. Among them, 29 residents had encountered patient suicide, with 12 directly involved. Symptoms of PTSD were detected in three residents. The physician directly involved in treating the suicidal patient reported the highest PCL-5 score. The majority of residents (27 out of 29) expressed the need for a structured support and training program tailored to healthcare professionals dealing with suicide.

**Conclusions:**

The findings suggest that psychiatric residents may require additional training and support to effectively address the complex issue of patient suicide. Implementing specific training programs could significantly enhance their ability to manage such situations.

**Disclosure of Interest:**

None Declared

